# Viewpoint: Patient safety in primary care – patients are not just a beneficiary but a critical component in its achievement

**DOI:** 10.1097/MD.0000000000035095

**Published:** 2023-09-15

**Authors:** Kevin T. Kavanagh, Lindsay E. Cormier

**Affiliations:** a Health Watch, Lexington, KY; b Health Watch, Eastern Kentucky University, Lexington, KY.

**Keywords:** CAHPS, culture of safety, EPA, just culture, Patient Reported Outpatient Safety Survey, patient survey, primary care, PROSS, SafeQuest, SAQ, SCOPE

## Abstract

Promoting and maintaining patient safety in primary care requires different strategies and monitoring than utilized in large healthcare delivery systems. Maintenance of a culture of safety is key to providing patient safety but has been difficult to measure in primary care. This is particularly true in rural settings where practice size is a major barrier to measurement reliability.

Primary care evaluates a wide range of patients, including those who are immunocompromised and others who have infectious diseases. Providing a safe environment with proper wearing of N95 masks, clean examination rooms, and adequate ventilation is important. Patients with infectious diseases should be separated from other patient populations.

Primary care is often less bureaucratic than hospitals, but also has fewer resources to implement patient safety initiatives, along with detecting safety lapses and adverse events. However, monitoring the practice’s safety practices and the culture of safety is of utmost importance and should be performed using both outcome and process measures. Because of the small size of many rural practices, effective monitoring of adverse events and maintenance of safety protocols should include patients. Patients are an important resource for reporting of adverse events and medical treatment outcomes.

The aim of this manuscript is to underscore the importance of patient safety in primary care and to stimulate future research in developing a metric for the culture of safety in primary care, which also incorporates the patient perspective. Patients should be viewed not only as beneficiaries of patient safety but also as a critical component of its maintenance.

## 1. Introduction

Maintaining patient safety in healthcare delivery systems has been a major concern during the COVID-19 pandemic. Most of our experience and research data has come from inpatient settings. During the prepandemic calendar year of 2018, Bates et al reported in the New England Journal of Medicine that almost 1 in 4 hospital admissions had an adverse event, 22.7% of which were deemed preventable (6.8% of all admissions).^[1]^ This is a dismal statistic, one that has changed little in over a decade from the findings of Classen, et al,^[2]^ where adverse events were found to occur in one-third of all hospital admissions, and confirmed by the findings of Landrigan et al,^[3]^ who reported 25 patient harms per 100 admissions. Antibiotic-resistant bacteria and fungi have flourished during the COVID-19 pandemic. According to the CDC,^[4]^ “COVID-19 (has) created a perfect storm” with hospital infections and deaths increasing by 15%. However, as illustrated with methicillin-resistant *staphylococcus aureus* hospital acquired infections,^[5]^ it is possible to maintain prepandemic levels of hospital-associated infections while maintaining adequate staffing and mitigation procedures, including active surveillance and contact precautions.

Even less is known about safety in primary care, a branch of medicine which has even less resources than hospitals to ensure patient safety. In primary care, the culture of safety has been largely ignored, with little published on the subject. In 2016, Verbakel et al,^[6]^ conducted an extensive literature search on this subject in the Journal of Patient Safety and found only 2 articles eligible for analysis. One described the role of an electronic medical record, and the other described the impact of 2 workshops on patient safety.

Primary care patient safety is not an easy subject to address because the specialty cuts across a myriad of different settings and practice environments. Those who practice in large facilities or groups are part of an institutional culture which may have similarities to inpatient settings. However, regardless of the setting, a culture centered on safety is of utmost importance.

Patient safety is of paramount importance in primary care. This report aims to outline the need for and to stimulate future research and development of a metric that measures the culture of safety in primary care and incorporates the patient perspective.

## 2. Small practice – primary care

This commentary focuses on small practices, less than 10 providers. These practices are often in rural settings. When the lead author first opened his small-town practice, it was common for employees to have multiple jobs and roles in the community. The practice’s business loan officer at the local bank was also the practice’s after-hours employee who was responsible for room cleaning.

In these settings, the office’s medical providers and personnel often assume multiple roles to assure patient safety. Small practices have little bureaucracy but also meager resources. In this environment, a number of major factors and domains require the maintenance of best practices to ensure optimal patient safety. The following points are discussed in this report:

1. Infectious disease safety during the COVID-19 pandemic and infectious disease outbreaks.
2. Efficient communication with patients.
3. Reporting of adverse events, complaints, and unexpected outcomes.
4. Monitoring of best practices.

### 2.1. Infectious disease outbreaks

Primary care, unlike most other specialties, evaluates and treats a wide range of patients, including those with highly communicable infectious disease. The spread of disease between patients in waiting rooms and offices is often all but ignored. The lead author’s 90-year-old mother recently visited her family physician for a leg wound and left with seasonal influenza that required hospital monitoring.

Understanding the spread of disease is critical. Do not think of “droplets” but instead of particles of various sizes that can become airborne and of surfaces where pathogens can reside for extended periods of time.^[7]^

Spread by surfaces is of utmost importance. For example, bacteria, such as methicillin-resistant *staphylococcus aureus*, can persist on surfaces for months.^[8]^ Proper cleaning of the office with appropriate disinfectants requires trained environmental staff with access to appropriate cleaning agents.

Aerosols are also important. Some pathogens aerosolize only if an infected patient undergoes an “aerosolizing” procedure. Unfortunately, SARS-CoV-2 is airborne and does not require a medical procedure to spread.^[9]^ The CDC warns that even talking^[10]^ and breathing^[11]^ can spread the virus.

Primary care often treats high-risk immunocompromised patients. Whether it is SARS-CoV-2 or the common cold, both conditions can be fatal. Ensuring that your office has safe air to limit the spread of aerosolized pathogens is of utmost importance. A glass barrier or even being in another room will not stop the spread of airborne diseases.

Central Heating, Ventilation, and Air-Conditioning (HVAC) systems should utilize a High Efficiency Particulate Air filter (HEPA) with a Minimum Efficiency Reporting Value (MERV) of 15, which can provide an approximately 80% decrease in airborne particles.^[12]^ The American Society of Heating, Refrigerating and Air-conditioning Engineers (ASHRAE) recommends that emergency room and radiology waiting areas have a minimum of 12 complete air exchanges per hour.^[13]^ The same should hold true for outpatient healthcare settings but this will vary depending on the occupancy.

A screening tool for adequate ventilation is the measurement of Carbon Dioxide (CO2) levels.^[14]^ Several portable monitors are available through the Amazon and are increasingly being used by the public.^[15]^ The outside CO2 level is approximately 400 ppm. The indoor levels should be maintained below 700 ppm. The American Society of Heating, Refrigerating and Air-conditioning Engineers recommends a maximum steady-state indoor CO2 level of approximately 870 ppm.^[16]^ Recommended ventilation rates depend on occupancy, with a minimal rate of 10 L per second per person.^[16]^ This rate has also been found to provide 80% mitigation of SARS-CoV-2 in classrooms,^[17]^ and high CO2 levels can also cause drowsiness and affect concentration.^[18]^ It is reasonable to set a ventilation goal below 700 ppm.

Installation of upper room Ultraviolet C germicidal lighting has been used for over 70 years and is an economical low maintenance method for improving air quality.^[19]^ It can achieve a high level of equivalent complete air exchanges and is an effective option for older HVAC systems that cannot support a MERV 15 filter. Just one of these systems may have avoided the lead author’s mother’s hospitalization for seasonal influenza and at a fraction of the cost.

During times of high viral spread, all office personnel should wear N95 masks. Despite the abundant misinformation regarding masking,^[20]^ these masks will mitigate spread of airborne pathogens by both filtration and an electrostatic effect.^[21]^

For high-risk patients, setting aside time in the morning should be considered for mandatory masking and evaluation. If the office is running behind schedule, give patients the option of waiting in their car to limit exposure to infected individuals and call them on their cell phones when they are ready to be seen. If possible, patients with communicable diseases should be in a separately ventilated area of the office. If this is not possible, they should be scheduled as the last patients of the day. Finally, it is necessary to frequently disinfect the office to eliminate spread by surfaces.

### 2.2. Efficient communication with patients

Communication with patients is of utmost importance and needs to go far beyond handing them a printed information sheet regarding their treatment. Marvel et al,^[22]^ found that in almost 25% of visits physicians never asked for an initial statement of patient concerns and the patient was able to complete their statement in only 28% of visits. In addition, patients only remember about 50% of the facts that are communicated to them.^[23]^ Health literacy can make effective patient communication more difficult in practices that service economically disadvantaged populations and patients whose native language is not English.

Adequate communication also includes ready patient access to prompt appointments, prescription refills and referrals.

Patients should also have ready access (both provided in printed form and online) to their current medications and dosages, along with a list of their medical conditions. If an emergency occurs, this will enable patients to immediately provide this information to other providers.

Written educational materials are not effective for some patients. Have an option to view a practice-produced video online or in the office. All materials should be available in additional languages as required by the patient demographics the practice services. A teach-back technique can enhance patient understanding of instructions by having the patient repeat back what they are told.^[24]^

Patients should be provided with treatment instructions after a procedure, along with face-to-face communication to answer questions. Patients must know how and who to contact in case of an emergency. A reliable on-call service and coverage should be provided after hours. During the day, physician extenders should be available to evaluate the patient and promptly answer patients’ questions and concerns.

### 2.3. Reporting of adverse events, complaints and unexpected outcomes

Panesar et al,^[25]^ have reported that between 2 to 3 safety incidents occur per 100 primary care encounters. Patients need to know where to obtain prompt help with adverse reactions to drugs and treatments. They also need to know ahead of time the early signs of complications and how to perform first aid. Surgical and procedural complications can be life threatening, and a fast medical response is crucial.

Whether you are a solo practitioner or a partner in a practice, it is important to know when adverse events and near misses occur, so that safety lapses can be corrected to prevent future reoccurrences. In addition, when harm occurs, an apology along with rapid disclosure and compensation should be provided to the patient.^[26]^ Most adverse events are not officially reported by practitioners or medical staff. Classen et al,^[2]^ found that voluntary reporting missed 90% of adverse events. Underreporting is not just a problem in the United States. Öhrn et al,^[27]^ analyzed 113 malpractice claims in Sweden that involved patient death or serious injury. Only 20% of cases were reported by medical officers as sentinel events to the National Board of Health and Welfare.

Zhu et al,^[28]^ conducted a large survey of patients regarding “negative effects” of hospital care. Negative effects were reported by 29% of 2582 patients. 71% of these cases were classified as adverse events after physician review. The authors concluded that patient reports are an important part of the incident-detection process.

Weingart et al,^[29]^ interviewed 228 adult patients; 8% experienced an adverse event and 4% experienced a near miss. However, only 55% of the adverse events and 31% of the near misses were found in the patient’s medical record, and none were reported in the hospital’s incident reporting system. Weissmann et al,^[30]^ compared post hospital discharge patient interviews with their medical records. Twenty-three percent of 998 patients reported at least 1 adverse event but only 11% had an adverse event recorded in their medical records. Khan et al,^[31]^ surveyed 383 parents of hospitalized pediatric patients and 8.9% reported safety incidents. Only 57% of the errors were documented in the patient’s medical record. Similar to other authors, Khan et al, concluded that parents frequently reported errors which were not otherwise documented, and that consideration should be given to incorporating family reports into adverse event surveillance systems.

The overriding consensus is that provider and staff voluntary reporting systems capture only a fraction of events and underscore the need for patient involvement in the reporting of adverse events and near-misses. Patient reporting can be facilitated by periodic surveys. Several researchers have found that this is a successful strategy. However, confidential reports are more likely to contain detailed codable information.^[32]^

Once an incident is reported, an investigation with feedback to patients and staff important. Root cause analysis should be undertaken with a systemwide review, not aiming to blame staff but to evaluate, detect and correct any system level problems.

The maintenance of a Just Culture is also important.^[33]^ Practices should implement a non-punitive strategy to facilitate adverse event reporting. Adverse events should undergo root cause analysis and be dealt with using the principles of Just Culture.

However, in small practice settings, the buck often stops with the physician as opposed to an administrator in a large institution. Thus, system problems often persist or corrected at the discretion of primary care practitioners. Work pressure on staff, training, procedures, and workloads are additional important factors regardless of the size of the practice.

## 3. Monitoring of best practices

### 3.1. Patient reported outpatient safety survey

The Agency for Healthcare Research and Quality (AHRQ) has several Surveys on Patient Safety Culture (SOPS) which are available for hospitals, nursing homes and outpatient medical offices.^[34]^ These surveys can be provided to office staff and providers.

The major domains of the medical office survey are as follows:

▪ Communication about errors.
▪ Openness of communications.
▪ Office Processes and Standards.
▪ Organizational Learning.
▪ Owner and Management Leadership.
▪ Perceptions of Patient Safety and Quality.
▪ Patient Care Tracking and Follow-up.
▪ Staff Training.
▪ Teamwork.
▪ Work Pressure.

A comprehensive 2018 systemic review of the literature by Lawati et al,^[35]^ found 28 papers that described surveys for primary care safety culture. The most commonly used survey instrument was the AHRQ Hospital Survey on Patient Safety Culture. However, this instrument may not be entirely suitable for small practice settings.

In 2018, Vasconcelos et al^[36]^ published a review of survey instruments used to evaluate the culture of safety in primary care. They identified 7 instruments, but only 3 of them were specific to primary care. The surveys were the Safety Culture Questionnaire for General Practice (SCOPE),^[37]^ the European Practice Assessment (EPA),^[38]^ and the Primary Care SafeQuest Safety Climate Survey (PC-SafeQuest).^[39]^

SCOPE is a Dutch adaptation of the AHRQ’s Hospital Survey of Patient Safety Culture and lacks many of the needed questions specifically designed for primary care patient safety. The EPA contains several process-related questions regarding primary care practices. Unfortunately, many of its questions and areas of emphasis do not reflect current medical practice. For example, the EPA has large sections on the security, content, and confidentiality of paper files, rather than electronic medical record systems. Questions regarding using a computer for referral letters, doctor’s access to emails and internet, along with contents of doctor bags, and a patient’s ability to request a home visit appear to no longer be areas in need of monitoring or of primary importance in today’s primary care settings. Texting has since evolved into one of the major modes of communication between a practice and patients. The EPA also has a financial domain and requirements for staff contracts and job descriptions that appear to have little relationship to patient safety. The PC-Safequest survey has generalized outcome measures and 5 domains: Workload, Communication, Leadership, Teamwork, and Safety.^[39]^ Bell et al found that PC-Safequest was poor at discriminating the reported safety climate between practices. The tool did not meet acceptable levels of reliability with a typical size practice comprising 11 responding staff.^[40]^

One of the most internationally used and studied instrument to measure safety culture is the Safety Attitudes Questionnaire (SAQ).^[41]^ The SAQ was developed by the University of Texas and funded by the Agency for Healthcare Research and Quality, the Robert Wood Johnson Foundation, and the Gottlieb Daimler and Karl Benz Foundation. In 2019, de Souza et al,^[42]^ modified the SAQ and described the use of the Safety Attitudes Questionnaire – Ambulatory Version (SAQ-AV). This 62 item questionnaire^[43]^ was also developed at the University of Texas and contains the following domains:^[43]^

▪ Teamwork climate
▪ Safety climate
▪ Perception of management
▪ Job satisfaction
▪ Working conditions
▪ Stress recognition scale
▪ Ambulatory process of care
▪ Other categories related to a large range of items (27) include the occurrence of errors and error reporting.

The SAQ-AV survey also appears to be related to a large practice setting. However, even small practice settings have developed cultures that can affect their practice. Because of the absence of oversight, which exists in large institutions, small practices can sometimes be susceptible to national politics, which can influence patient safety. Physicians may abandon or not promote mainstream science, as illustrated by some providers recommending the use of ivermectin for COVID-19 and the shunning of vaccines, placing patients in harm’s way.

Obtaining a patient’s perspective regarding the safety cultures and experiences of the practice is also important. The Agency for Healthcare Research and Quality (AHRQ) has developed a Consumer Assessment of Healthcare Providers and Systems Clinical and Group Survey.^[44]^ This survey records patient experiences in the following domains.

- Accessibility of care,
- Communication with providers,
- Care coordination, and
- Interactions with staff

The survey can be downloaded from: https://www.ahrq.gov/cahps/surveys-guidance/cg/index.html.

Although this AHRQ survey has several domains related to communication, accessibility to care, and staff interactions it is not centered on observed safety practices and adverse event reporting.

Primary care, more than any other specialty, centers on patients. The patient delivery hierarchy may be as simple as Doctor-Nurse-Receptionist-Patient. Patients more than any other individual in the hierarchy must be involved in the evaluation and assurance of safety, along with providing insights into the practice’s culture of patient safety. In addition, the incorporation of patients into the survey process will allow a larger “n” and increase a survey’s reliability for small practices, possibility eliminating a reliability problem similar to the one encountered with PC-Safequest.

There have been extensive measurements of healthcare outcomes by patients (Patient-Reported Outcome Measurements or PROM)^[45]^ and computerized methodologies have been developed that employ strategies for measuring patient reported quality of delivered care.^[46]^

However, patient surveys emphasizing the safety of the healthcare received by patients are lacking. There are numerous patient satisfaction surveys, but there is little agreement regarding their composition.^[47]^ A Hasting study has described 3 main components.^[48]^ These include essential medical care, treatments sought by patients and families, along with provider activities and behaviors related to compassionate care.

Unfortunately, patients are viewed more as beneficiaries of patient safety, rather than being recognized as a critical component in its maintenance. An instrument specifically created from the ground-up for primary care, such as the EPA, but with updated domains and questions designed for surveying patients, is needed. The proposed components of the patient survey are listed in Table 1. Periodic surveillance of patients to identify safety lapses, adverse events, staff overload, and quality of the practice environment may greatly aid in establishing and maintaining a culture of safety in primary care.

**Table 1:** Domains and potential questions for patient reported outpatient safety survey.

1. Access to care -- Were you able to obtain your appointment in a timely fashion? -- If you received a referral, were you able to obtain one quickly? -- Did you have to forgo or delay testing, medications, or treatments because of cost? -- Did your provider discuss lower cost drug or treatment alternatives with you? 2. Communication regarding care -- Is there a system for you to anonymously report a practice concern or adverse event? -- Can you contact the practice off hours to ask medical questions? -- Were risks of new treatments explained in a way you understood them? -- Did you have enough time during your visit to ask questions? -- If you had an office procedure, were you called the next day? -- Were you provided understandable information regarding care after your procedures? -- Does your provider provide you with an easily accessible list of your medications and medical conditions? -- Were you reminded of your appointment by E-Mail or text? -- Is your online medical record easy for you to access, understand and use? 3. Adverse events -- Did you experience a medical error? -- If you experienced an error did it limit your participation in daily activities? -- Were you able to obtain help in a timely manner for this event? -- Did you observe an unsafe practice during your visit? -- Were you given a prescription for medications you are allergic to or contraindicated with other medications you are prescribed? -- Did you receive a wrong diagnosis? -- If a procedure or major treatment was recommended, were you offered to obtain a second opinion? -- Did you feel you may have caught an infectious disease during your visit? 4. Observed safety -- Did you observe your provider wash his hands upon entering the examination room -- During times of high viral spread (RSV, influenza, COVID-19) did the staff wear masks? -- Was masking required by patients? -- Were patients with suspected infectious diseases separated from others? -- If the practice was behind schedule, were you given an option to wait in the car? -- Were you asked questions regarding exposure to infectious disease circulating in the community, such as seasonal influenza or COVID-19? -- If you are at high risk, is there a time set aside in the morning to be seen promptly with minimal exposure to other patients? -- Did you observe any debris or patient secretions (blood, etc.) in the practice setting? -- Did you feel “safe” from physical harm and from catching infectious diseases during your visit? 5. Workplace conditions and staff overload -- Was your time in the doctor’s examination room too short to take care of your problem? -- Were there long lines at the check in or checkout counter? -- When you call the office, are you placed on hold for a protracted period of time? -- Are your medication refill requests processed in a timely manner? -- Did the staff appeared hurried and not having enough time to complete their tasks?

Flu = influenza, RSV = respiratory syncytial virus.

## 4. Conclusion

Workplace protocols need to be implemented to establish a safe practicing environment, including the prevention of the spread of infectious diseases. This includes proper environmental cleaning and office ventilation to prevent the spread by surfaces and aerosols. Patient involvement in the identification of adverse working environments, communication difficulties, barriers to care, safety lapses, near-misses, and adverse events will provide critical information to improve safety practices. This would also be expected to increase both the quality and reliability of the survey. Once an adverse event is identified, the patient needs to be given prompt support, and a root cause analysis is performed utilizing the principles of a Just Culture.

The prevention of adverse events in primary care can be enhanced by maintaining a strong and effective culture of safety. Similar to other areas of healthcare, the measurement of the culture of safety is important. However, the size and organizational structure of many primary care delivery systems, especially in rural settings, make metrics that focus only on healthcare staff and providers less reliable. Future development of metrics that incorporate patients has the potential to increase reliability due to a large sample size and even increase in validity by incorporating another point of view. Patients should be viewed as a critical component for patient safety maintenance and promotion and not just one of the beneficiaries.

## Author contributions

**Conceptualization:** Kevin Kavanagh.

**Resources:** Kevin Kavanagh, Lindsay E. Cormier.

**Writing – original draft:** Kevin Kavanagh.

**Writing – review & editing:** Lindsay E. Cormier.
